# Antibodies induced by ancestral SARS-CoV-2 strain that cross-neutralize variants from Alpha to Omicron BA.1

**DOI:** 10.1126/sciimmunol.abo3425

**Published:** 2022-05-10

**Authors:** Ian W. Windsor, Pei Tong, Olivia Lavidor, Ali Sanjari Moghaddam, Lindsay G.A. McKay, Avneesh Gautam, Yuezhou Chen, Elizabeth A. MacDonald, Duck Kyun Yoo, Anthony Griffiths, Duane R. Wesemann, Stephen C. Harrison

**Affiliations:** ^1^ Boston Children’s Hospital, Boston, MA 02115, USA; ^2^ Department of Biological Chemistry and Molecular Pharmacology, Harvard Medical School, Boston, MA 02115, USA; ^3^ Ragon Institute of MGH, MIT, and Harvard, Cambridge, MA 02139, USA; ^4^ Department of Medicine, Division of Allergy and Clinical Immunology, Division of Genetics, Brigham and Women’s Hospital, Harvard Medical School, Boston, MA 02115, USA; ^5^ Department of Microbiology, Boston University School of Medicine, Boston, MA 02115; ^6^ National Emerging Infectious Diseases Laboratories, Boston University, Boston, MA 02115; ^7^ Massachusetts Consortium on Pathogen Readiness, Boston, MA 02115, USA; ^8^ Howard Hughes Medical Institute, Boston, MA 02115, USA

## Abstract

Neutralizing antibodies that recognize the SARS-CoV-2 spike glycoprotein are the principal host defense against viral invasion. Variants of SARS-CoV-2 bear mutations that allow escape from neutralization by many antibodies, especially those belonging to classes widely distributed in the human population. Identifying antibodies that neutralize these variants of concern and determining their prevalence are important goals for understanding immune protection. To determine the Delta- and Omicron BA.1-variant specificity of B cell repertoires established by an initial Wuhan strain infection, we measured neutralization potencies of 73 antibodies from an unbiased survey of the early memory B cell response. Antibodies recognizing each of three, previously defined, epitopic regions on the spike receptor-binding domain (RBD) varied in neutralization potency and variant-escape resistance. The ACE2 binding surface (“RBD-2”) harbored the binding sites of the neutralizing antibodies with highest potency but with the greatest sensitivity to viral escape; two other epitopic regions on the RBD (“RBD-1 and “RBD-3”) bound antibodies of more modest potency but greater breadth. The structures of several Fab:spike complexes that neutralized all five variants of concern tested, including one Fab each from the RBD-1, -2 and -3 clusters, illustrated the determinants of broad neutralization and showed that B cell repertoires can have specificities that avoid immune escape driven by widely distributed (“public”) antibodies. The structure of the RBD-2-binding, broad neutralizer shows why it retains neutralizing activity for Omicron BA.1, unlike most others in the same public class. Our results correlate with real-world data on vaccine efficacy, which indicate mitigation of disease caused by Omicron BA.1.

## Teaser

Structures of SARS-CoV-2 spike-bound antibodies from Wuhan-strain infection show features needed to neutralize variants of concern.

## INTRODUCTION

The high global rate of SARS-COV-2 infections leads to regular emergence of new variants (*1*). Both transmissibility and immune evasion appear to determine fitness of variant phenotypes. Strength of attachment to the viral receptor, ACE2, is one determinant of transmissibility, restricting the variability of the receptor binding surface. But this restriction also limits the potential for escape from the most potent neutralizing antibodies, which share contacts with ACE2 and neutralize by blocking cell entry. That is, immune escape and receptor binding exert competing selective pressures on the evolution of the SARS-CoV-2 spike protein, which is both the viral receptor-binding and fusion protein and the sole known target of protective antibodies (Abs). Abs that maintain affinity to variants reflect the outcome of these countervailing selective forces.

We recently characterized cross-competition for spike binding by 73 monoclonal antibodies (mAbs) in an epitope-unbiased set representing sequences of memory B cell receptors (BCRs) from 19 COVID-19 convalescent donors (*2*). Clustering analysis of those competition data define seven principal clusters, corresponding to distinct epitopic regions on the SARS-CoV-2 spike. Three of those regions are on the receptor-binding domain (RBD), two on the N-terminal domain (NTD), and the rest on the S2 fragment. The panel of 73 mAbs is thus a broad sampling of components of the human polyclonal response to infection with a Wuhan-like (i.e., early pandemic) SARS-CoV-2.

The most potent neutralizing Abs, from many studies, fall into the RBD-2 competition group, which includes all that recognize epitopes overlapping the ACE2 footprint on the spike-protein RBD (*2*). Many of the RBD-2 Abs belong to well-studied public classes (*3*) (*4*–*12*)—that is, they have V(D)J recombined gene segments for both heavy and light chains and heavy-chain complementarity-determining regions (HCDR3s) of similar length and sequence in common with others found in many unrelated donors from diverse cohorts. Although more neutralizing mAbs in our unbiased set fall into the RBD-1 competition group than into RBD-2, their activity is generally weaker (*2*). Because it contains the target sites for potent neutralization, the RBD-2 epitopic region has varied more than RBD-1 and RBD-3, despite strong constraints on the receptor-binding surface. Most of the RBD-2 Abs maintain affinity and in vitro pseudotype neutralization potency for the alpha variant, but not for beta, or gamma.

We report here extension of our previous analysis to the delta and Omicron BA.1 variants. We identified one mAb in each group that binds and neutralizes all five variants tested here (alpha, beta, gamma, delta, and Omicron BA.1), as well as a fourth (in RBD-2) that neutralized all variants tested except Omicron BA.1. Each of the two RBD-2 competition-group members belonged to a public class. Most known members of one of these 2 public classes fail to neutralize beta, gamma, and Omicron BA.1; the RBD-bound structure of the exception described here suggests the basis for its broader neutralizing activity. The results provide a molecular rationale for flexibility in the antibody system to extend neutralizing recognition breadth from an ancestral strain across evolving viral variants (*13*–*15*).

## RESULTS

### Memory B cell repertoire contribution to SARS-CoV-2 variant neutralization

**Fig. 1.**
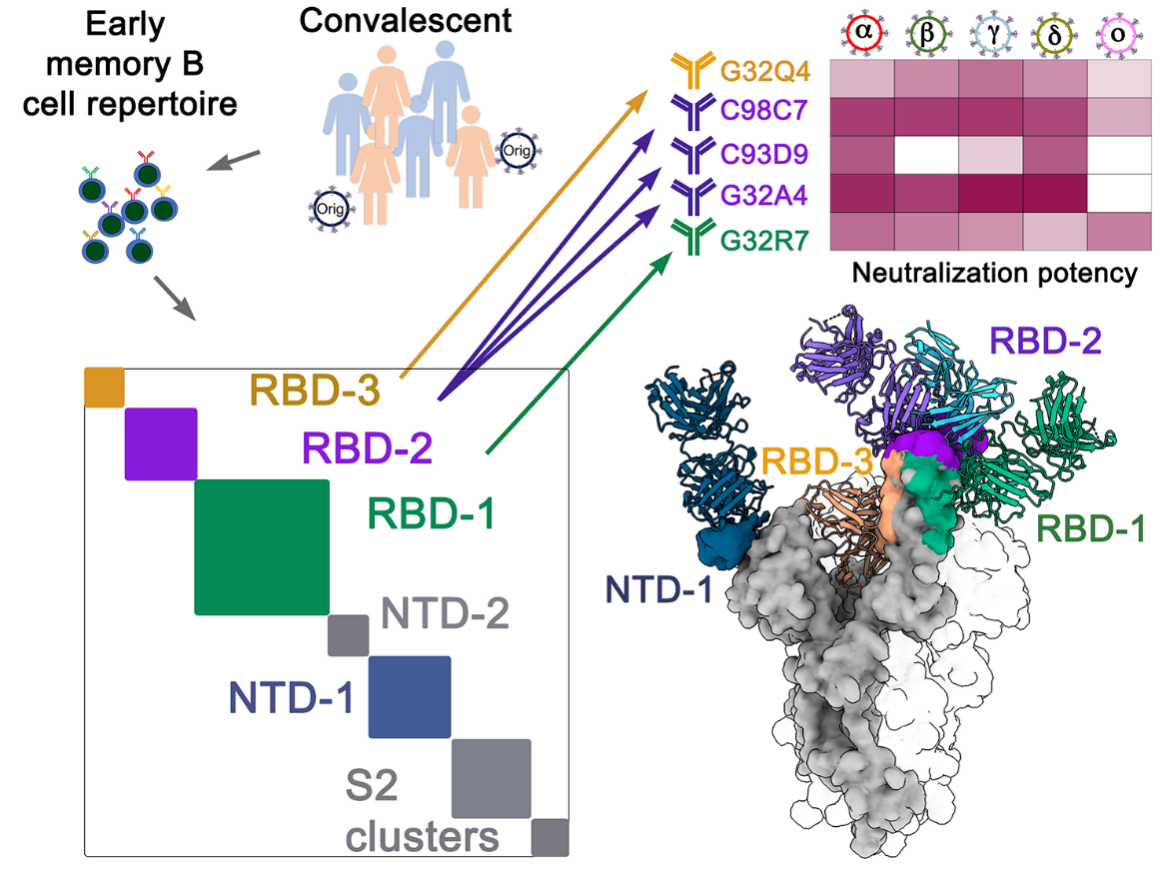
**Schematic diagram of the source of the 5 mAbs (G32Q4, C98C7, C93D9, G32A4 and G32R7) analyzed in this paper**. Seventy-three mAbs were expressed from single-cell, paired-chain BCR sequences of SARS-CoV-2 spike-positive memory B cells, sorted from 19 convalescent donors, and the corresponding epitopic regions on the spike protein were assigned by competition ELISA (2). The analysis yielded 7 clusters, RBD-1,-2 and -3, NTD-1 and -2, and S2-1 and -2, in designations derived from the domain the mAbs bind and the order of abundance, for each domain, of mAbs in the cluster. For each of the 73 mAbs, spike binding and neutralization potency were determined, for five variants of concern as well as for the D614G variant of the original Wuhan isolate. Structures of selected mAbs mapped 4 of the 7 the epitopic regions, represented by the colored blocks on the diagonal of the cluster plot and by the correspondingly colored patches on the surface rendering of one subunit in the molecular model, with other parts of the subunit molecular surface in gray. The structures permitted interpretation of the activities of these mAbs for each of the variants of concern.

The left-hand side of Fig. 1 illustrates our published characterization of the early memory B cell repertoires from 19 convalescent donors during the first 6-8 months of the pandemic and hence from infection with virus closely related to the original Wuhan isolate (*2*). The colored blocks in the schematic diagonal plot represent the 7 clusters from our all-against-all competition antibody-binding assays—three RBD-binding clusters, two NTD clusters, and two S2 clusters. We mapped four of the corresponding epitopic regions (RBD-1,-2, and -3 and NTD-1), by determining representative structures and by including Abs for published structures in the competition experiments. We also determined neutralization profiles of all 73 Abs for the Wuhan isolate and for the alpha, beta, and gamma variants.

**Fig. 2.**
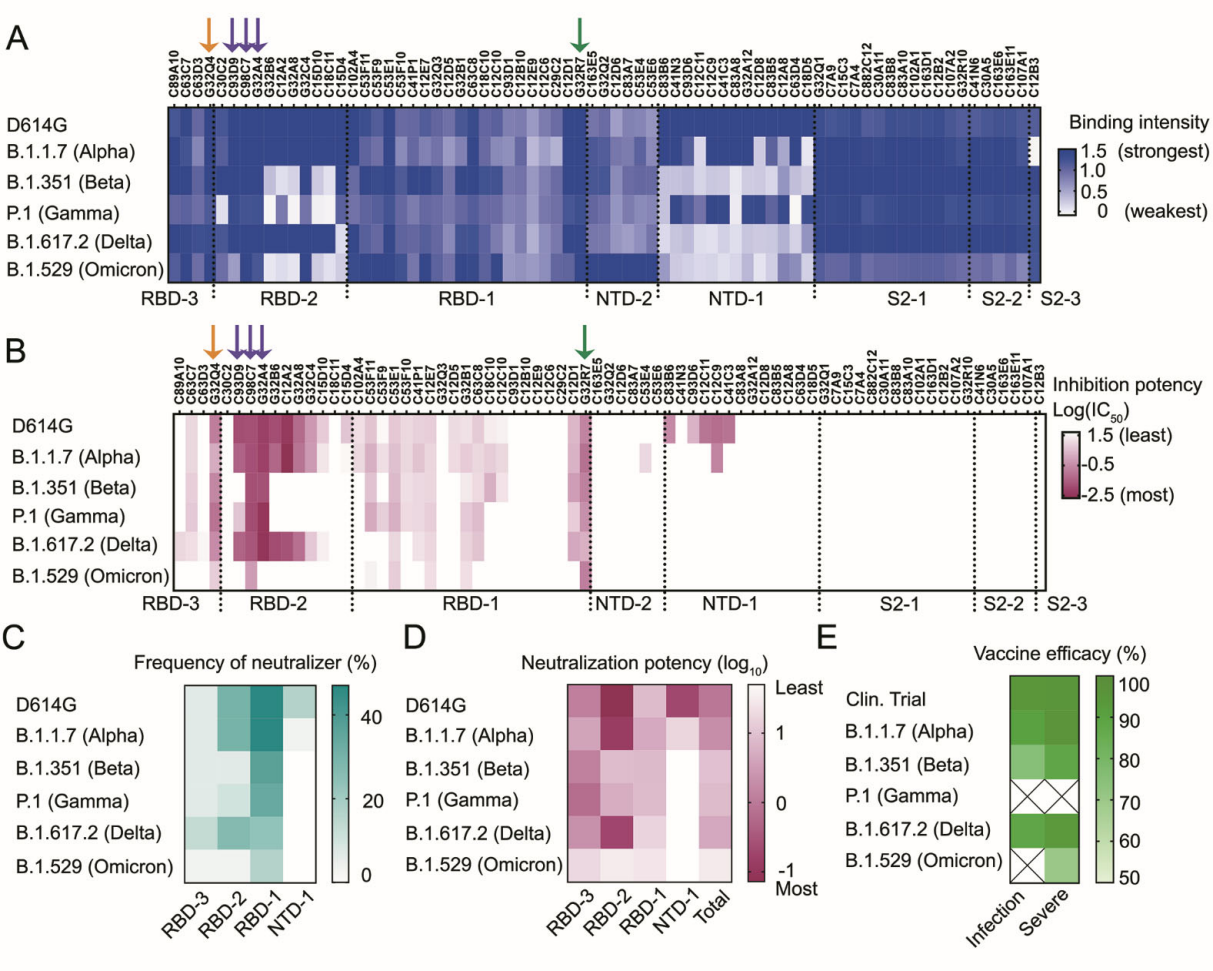
Binding and neutralization of the delta and Omicron BA.1 SARS-CoV-2 variants together with previously reported data for the alpha, beta, and gamma variants. (A) Binding heat map for the 73 mAbs with the delta and Omicron BA.1 variants; previously published binding to D614G, alpha, beta, and gamma variants shown for reference (2). Relative binding strengths in shades of blue. Arrows pointed the 5 mAbs in Fig. 1. All mAbs binding to delta or Omicron BA.1 variants were repeated at least once together with D614G. (B) Delta- and Omicron BA.1-variant neutralization potency heat map for the 73 mAbs; previously published neutralization of alpha, beta, and gamma variants shown for reference (2). Log_10_ IC_50_ (μg/ml) in shades of dark red. Values of IC_50_ are in Table S1. Arrows point to the 5 mAbs in Fig. 1. All mAbs were repeated at least twice with duplicated wells. (C and D) Percent of neutralizing mAbs (IC_50_ < 50 μg/ml) assigned to the indicated epitopic regions, unweighted (C), and weighted (D) by neutralizing potency (log_10_[IC_50_]). (E) Summary of clinical trial and real-world observational mRNA vaccine effectiveness against variants of concern with respect to total infections and severe outcomes (hospitalization, death).

In the work reported here, represented by the schematics on the right-hand side of Fig. 1, we examined binding and neutralization of the delta and Omicron BA.1 variants by mAbs in our panel. We characterized three antibodies that neutralized all five variants of concern tested (G32R7, C98C9, and G32Q4), one from each of the RBD clusters, as well as two others from RBD-2 (one that neutralized all variants except Omicron BA.1; the other, only alpha and delta). Figures 2A-D, show binding of each of the 73 mAbs to SARS-CoV-2 spike and their neutralization potencies for the D614G variant of the original Wuhan isolate and for the five variants of concern derived from it. Mutations in NTD-1 occur readily (*16*); they eliminated neutralization of all the variants by the NTD-1 Abs in our panel (Fig. 2B), and also substantially lowered affinity (Fig. 2A). The binding assay was more sensitive than the neutralization assay, accounting for the instances of detectable binding but undetectable neutralization. Three of the eleven RBD-2 competition-group mAbs in our panel (G32A4, C98C7, and G32C4) retained strong binding to all five variants of concern tested (Fig. 2A and Fig. S1) but only one of these (C98C7) neutralized all five, while a second (G32A4) neutralized all but Omicron BA.1. Like the majority of our RBD-2 mAbs, most clinical-stage mAbs compete with ACE2 and lose neutralization potency for Omicron BA.1 (*17*). Loss of the RBD-2 contribution to beta- and Omicron BA.1-variant neutralization in our assay correlated with escape of these variants from immune control of infection as monitored in studies (see Methods for specific references) of real-world vaccine effectiveness (Fig. 2E).

### Affinity of broad mAbs for variants of concern

Table 1 shows affinities of the antigen-binding fragments (Fabs) from mAbs G32R7, C98C7, G32A4, and G32Q4 for the RBDs of the D614G Wuhan-isolate mutant and of the variants of concern. Measuring the monomer-monomer interaction between a Fab and the monomeric RBD avoided format- and epitope-dependent avidity effects that might have confounded comparisons of specific mutations. The quantitative measurements correlated with neutralizing potency. Although all four Fabs bound the RBDs of variants alpha, gamma, and delta, as well as that of the unmutated Wuhan isolate, all but G32R7 lost affinity for the Omicron BA.1 RBD. The equilibrium dissociation constants for the C98C7 and G32Q4 Fabs (22 and 1.8 μM, respectively) were consistent with the diminished, but measurable neutralizing potencies of the corresponding mAbs. The Omicron BA.1 mutations eliminated G32A4 binding completely. The effects of mutations on affinity were consistent with the antibody footprints, as shown by the structures described below.

**Table 1.** Equilibrium dissociation constants for broad mAbs and RBDs and pseudovirus neutralization potency for coordinate variants of concerns.

	Original		Alpha		Gamma		Delta		Omicron BA.1	
mAb	*K*_d_	IC_50_	*K*d	IC_50_	*K*_d_	IC_50_	*K*_d_	IC_50_	*K*_d_	IC_50_
G32R7	< 1	0.109	3	0.080	22	0.375	50	1.851	68	0.162
G32Q4	57	0.578	75	1.476	13	0.089	53	0.316	1800	6.666
C98C7	3	0.013	6	0.015	1	0.012	3	0.023	22000	1.067
G32A4	53	0.014	60	0.007	360	0.003	290	0.003	n.b.	>50

Values of *K*_d_ reported in nM. n.b., no detectable binding

Values of IC_50_ are reported in μg/ml with 50 μg/ml as upper limit. IC_50_ of original, alpha, and gamma were reported previously (*2*).

### Cryo-EM structures of broadly neutralizing mAbs

**Fig. 3.**
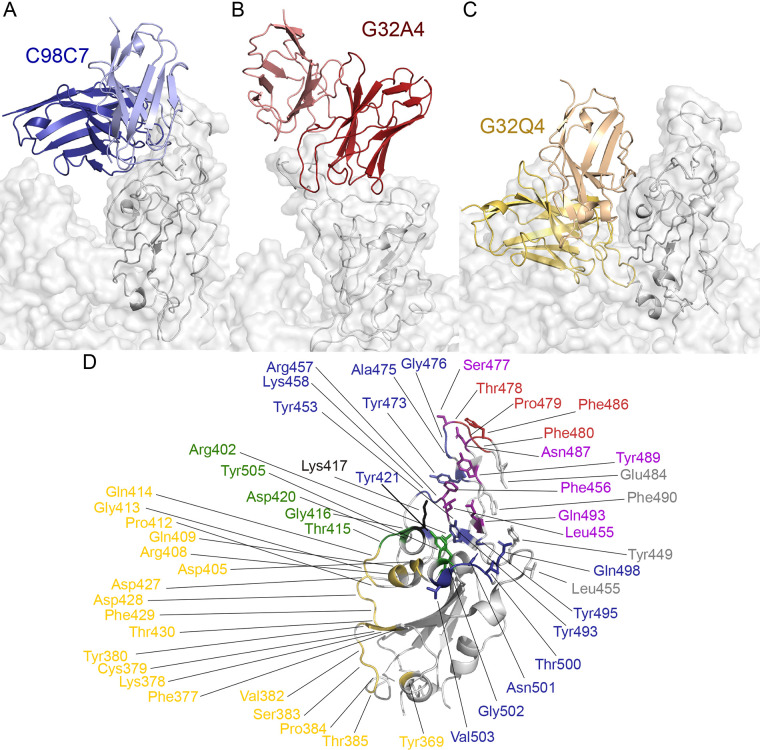
RBD-bound structures of cross-neutralizing antibodies. In all panels, the view is of the RBD in in the “up” configuration on a spike trimer with the threefold axis vertical. The RBD from each local reconstruction is aligned to the “up” RBD from the full spike (7krr) for reference, which is shown as a transparent surface. (A) Structure of C98C7 (blue, with light chain in a lighter shade than the heavy chain and only the Fv module shown) bound with RBD (dark gray). Light surface representation shows the outline of the spike. (B) G32A4 (red, with lighter shade for light chain and other details as in (A). The view is rotated with respect to all the other panels by about 60° ccw about the vertical. (C) As in (A), for G32Q4 (gold). (D) Cartoon representation of the RBD. The footprints of C98C7, G32A4, G32Q4 and and G32R7 (including side chains) are in blue, red, gold and green, respectively. Residues identified by PISA at the interface of the RBD and Fab are shown as sticks and their labels underlined.

**Fig. 4.**
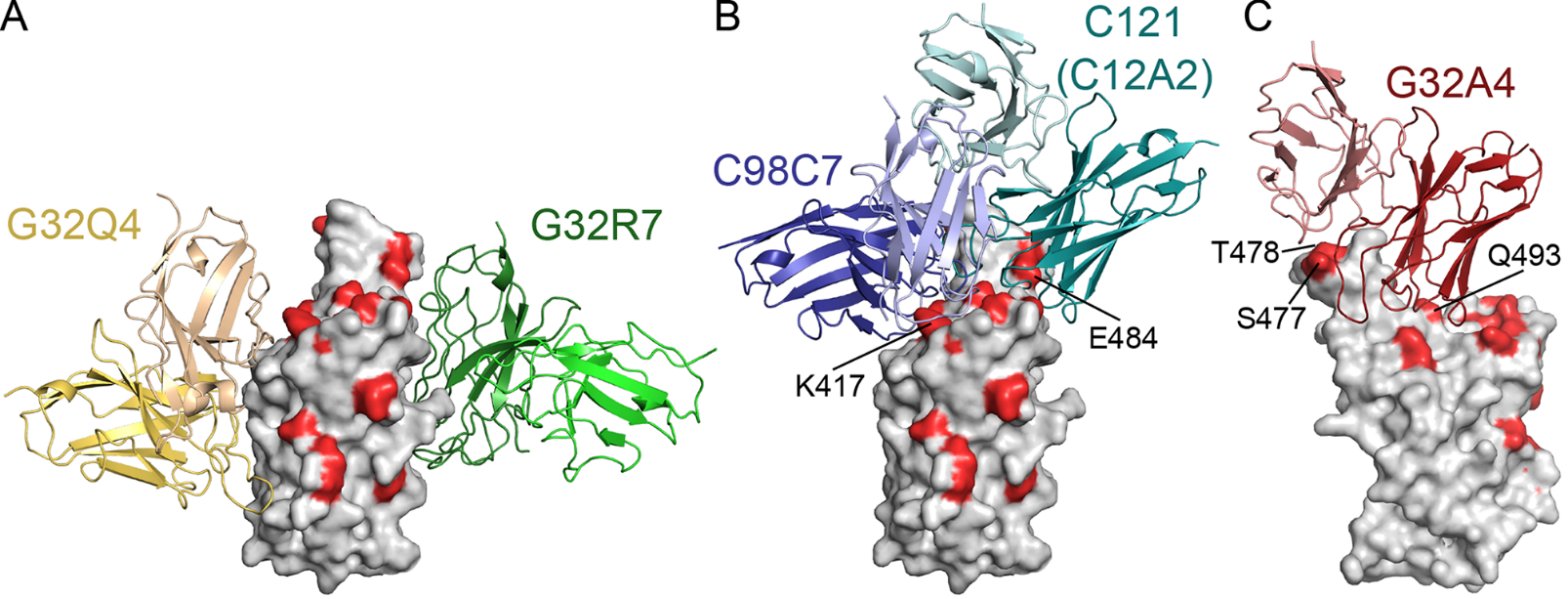
Relationship of cross-neutralizing antibody contacts to positions of residues mutated in the Omicron BA.1 variant. The RBD is in gray surface representation, with mutated residues in red. The Fv modules of the antibodies are in cartoon representation. (A) G32Q4 (gold) and G32R7 (green). These antibodies bind epitopic regions RBD-3 and RBD-1, respectively, on opposite faces of the domain. Orientation of view as in panel A of Fig. 3, with RBD as if in the “up” position on a trimer viewed normal to the threefold axis. (B) C98C7 (blue) and C12A2 (blue-green), with heavy chains encoded by IGVH3-53/66 and IGVH1-2, respectively. The very similar C121, for which a high-resolution crystal structure is available (*3*), was used to fit C12A2 (*2*). Viewed as in panel B in Fig. 3, with model rotated around the vertical axis by about 60° (ccw, looking down) with respect to its orientation panel A. (C) G32A4 (IGVH1-58). View as in panel A.

We determined structures for prefusion stabilized spike ectodomain trimer bound with Fab fragments of three mAbs (G32A4, C98C7, and G32Q4), two of which (C98C7 and G32Q4) neutralized all variants, while the third (G32A4) neutralized all except Omicron BA.1 (Figs. 3 and 4). Local reconstructions enabled us to obtain resolutions sufficient to model the antibody-antigen interaction (Figs. S2 and S3, Table S2). The epitopes of C98C7 and G32A4, both in the RBD-2 group, overlapped the ACE2 footprint (Fig. 3A and B); related Abs compete with ACE2 (*2*). G32Q4, in the RBD-3 cluster, bound the face of the RBD that contacts an adjacent RBD in the “down” conformation; it competed with CR3022, an antibody originally isolated from a convalescent SARS-CoV infected donor (Fig. 3C and (*18*)).

G32A4 and C98C7 belong to well-studied public classes, which dominate the RBD-2-cluster (Table S3 and (*2*)); G32Q4 showed a convergence of structure and recognition with a published antibody encoded by completely different V_H_ and V_L_ gene segments. C98C7, a product of V(D)J recombination of IGVH3-53/66 with IGHD3-22 and IGJH6, is nearly identical in the sequences of both variable domains to antibody P2C-1F11 (*19*). Despite this similarity, the structure showed that the pose of the C98C7 Fv module with respect to the RBD deviated slightly from that of P2C-1F11, perhaps because of a glycine residue rather than a serine at position 30 of the light chain (Fig. S4A). Its heavy-chain contacts were almost identical to those of P2C-1F11, but the light chain contacted the RBD more intimately, especially near residue 501, correlating with different main-chain configurations for LCDR2 (Fig. S4A). G32A4, with a heavy chain encoded by IGVH1-58, bound the RBM with contacts like those of other members of its public class (Fig. S4B) (*9*–*12*). The G32Q4 heavy chain derives from V(D)J recombination of IGVH3-30, one of the most prevalent heavy chain V gene segments in humans (*2*). Although there are published structures for several spike-directed IGVH3-30 Abs (*5*, *20*, *21*), none reported so far share an epitope with G32Q4. Instead, we found the HCDR3 loop of G32Q4 to be nearly identical to that of COVA1-16 (*22*), both from recombination of gene segments IGHD3-22 and IGHJ1, while the COVA1-16 heavy chain has an otherwise very different sequence encoded by IGHV1-46. The two Abs have nearly identical RBD recognition patterns (Fig. S4C and (*22*)).

### Variant mutations and breadth of neutralization and binding

One antibody from each of the three RBD epitopic regions (G32R7, C98C7, and G32Q4, in competition clusters RBD-1, -2 and -3, respectively) neutralized all five variants of concern in a pseudovirus assay (Fig. 2); the same three also neutralized authentic SARS-CoV-2 Omicron BA.1 (Fig. S6). The spike-bound structure of the G32R7 Fab bound the face of the RBD that is adjacent to the NTD when the RBD is in the down conformation (*2*). Its footprint included none of the Omicron BA.1 RBD mutations. G32R7 does not belong to a recognized public class, and its contacts with the RBD included many from its long (24-residue) HCDR3. Three other RBD-1-class mAbs also had detectable neutralization activity for all variants we tested (Fig. 2B and Table S1), but their potency was low, even for the D614G parent strain, despite relatively high apparent affinities.

The observed footprint of antibody G32Q4 avoided any of the Omicron BA.1 variant mutations (Fig. 4A). In addition to the convergence of its HCDR3 sequence and RBD interface with that of COVA1-16, the divergent LCDR2 loops of both these Abs contacted the RBD between residues 405 and 410. For G32Q4 to bind, both the targeted RBD and the adjacent RBD need to be in the “up” configuration. The immune-escape mutations in the beta, gamma, and Omicron BA.1 variants fell within the footprint of RBD-2-cluster antibody C98C7. Nonetheless, unlike other Abs with heavy chains encoded by IGHV1-53/66, such as C93D9 (see (*2*)), C98C7 retained some binding and neutralizing potency for all the variants, including Omicron BA.1 (Figs. 2B and 4B).

VH1-58 Abs, including G32A4, bind and potently neutralize all the variants of concern except Omicron BA.1 (*23*–*26*). Several Omicron BA.1 mutations fell within the epitope of G32A4 (Fig. 4C). One of them, Q493R, which contributes to ACE2 affinity through a salt bridge (*27*, *28*), appeared as an escape mutant in a selection for escape from neutralization by antibody B1-182.1, a member of the VH1-58 public class (*9*, *29*, *30*). Two others are S477N and T478K. The latter, also present in delta, had no measurable effect on binding and neutralization of delta by the four Abs studied here and thus could not alone explain the loss of affinity for Omicron BA.1. To assess the effects of the other RBM mutations, we measured binding of the S477N/T478K and Q493R mutant RBDs with Fab G32A4 and found ratios, to binding the Wuhan-isolate RBD, of 15 and 70 for their equilibrium dissociation constants (Table S4, Fig. S5). The structure of the G32A4 complex showed that, barring local main-chain rearrangements, the arginine side chain of the mutant would overlap the antibody HCDR2. Loss of interaction between D100c in VH1-58 mAbs and S477 (Figure S4B) may also contribute to reduced affinity of the G32A4 Fab for the Omicron BA.1 RBD.

IGHV1-2 encodes the heavy chains of another public antibody class whose members lose affinity for beta and gamma (Fig. 4B) (*31*). These Abs contact the sidechain of E484 (*31*), mutated to lysine in the beta and gamma variants; these two variants lose sensitivity to IGHV1-2 Abs as well as to certain IGHV3-53/66 mAbs that bind with a different footprint from the majority (*32*). The E484A mutation in Omicron BA.1 apparently also compromises binding of IGHV1-2 Abs, such as C12A2 (Figs. 2A and 4B), by loss of either bulk or negative charge, even without the charge reversal found in beta and gamma. Abs from each of the well-characterized public classes thus contact at least one of the few sites of mutation–K417 and E484 in particular–found in multiple variants of independent origin.

## DISCUSSION

Three VH gene segments, IGHV1-2, 1-58, and 3-53/66, account for seven of the eleven RBD-2 mAbs in our unbiased sampling of nineteen convalescent donors (*2*). The structures we report here and in our previous work (*2*) confirm that their interactions conform to the stereotypical contacts seen in published structures of Abs in the corresponding public classes. The positions of mutations leading to resistance that have appeared in distinct viral lineages are primarily within the footprints of IgGs such as C98C7, G32A4, and C12A2, consistent with the expectation that widespread occurrence of such Abs will likely give them a prominent role in selecting for viral immune escape mutations. The recurring substitution K417N/T, present in the RBDs of the beta, gamma, and Omicron BA.1 variants, greatly reduces the affinity of most IGHV3-53/66 Abs for the SARS-CoV-2 RBD, and the E484K substitution, also present in beta, gamma, and Omicron BA.1, reduces the affinity of most IGHV1-2 Abs. No repeatedly found mutations lie within the footprints of the more “idiosyncratic” G32R7 and G32Q4, in the RBD-1 and RBD-3 competition groups, respectively. The several other Abs reported to neutralize all five variants of concern (*17*, *33*) that are not members of any identified public class. Together with G32R7 and G32Q4, they may represent responses not prevalent enough to have been major determinants of antigenic drift. Collectively, such broadly protective Abs might be relatively widespread.

C98C7 is unusual among RBM-binding, IGHV3-53/66 Abs in retaining affinity for the Omicron BA.1 variant spike protein. A characteristic of this public class is a short HCDR3 loop projecting toward Lys417, the site of the K417N/T mutation common to beta, gamma, and Omicron BA.1. Third hypervariable loops longer than about 11-12 residues would probably collide with the RBD if the antibody docked as defined by its germline encoded HCDR1 and HCDR2. Spike-binding, IGVH1-53/66 Abs bearing a non-polar residue at the tip of HCDR3 frequently pair with light chains encoded by IGKV1-9; those with an acidic residue at the tip of HCDR3, which can salt-bridge with Lys417, tend to pair with IGKV3-20 (*8*). The HCDR3 of C98C7 is non-polar, but it pairs with a light chain encoded by IGVK3-20; antibody P2C-1F11, which like C98C7 has a non-polar residue at the apex of HCDR3 but pairs with IGVK3-20 (*8*), is also insensitive to the mutations in the beta and gamma variants (*2*, *8*). Their structures suggest that interactions of the IGVK3-20 LCDR1 with the RBD loop that surrounds residue 501 (mutated from Asn to Tyr in all five variants) might compensate for any loss of affinity from heavy-chain contacts due to the K417N mutation, consistent with a published light-chain swap experiment with IGHV3-53 Abs (*34*).

G32A4, like other IGVH1-58 Abs, is insensitive to the K417N/T and E484K mutations in the beta and gamma variants and to T478K in delta, but it fails to neutralize Omicron BA.1, probably because of the S477N and Q493R substitutions. It nonetheless has detectable binding to Omicron BA.1, suggesting that just a few amino-acid differences could restore neutralizing potency. The sequence variations seen in clonal lineages of human Abs elicited by exposure to influenza virus (*35*, *36*) suggest that the amino-acid differences required might indeed be present in the memory B cell repertoires of many individuals. Reactivation of that B cell memory could have a role in protection against developing severe disease.

The N501Y mutation, common to the alpha, beta, gamma, and Omicron BA.1 variants, but absent in delta, increases ACE2 affinity. The K417N/T mutation, present in beta, gamma, and Omicron BA.1, decreases it (*37*). Thus, RBM mutations in the beta and gamma variants allowed them to escape neutralization by Abs common to the repertoires of donors in many cohorts, such as those specified by IGHV3-53/66 and IGHV1-2, but at the cost of affinity for ACE2. Alpha and delta spread rapidly and remained until displaced by other variants: they were thus in evolutionary terms more successful than beta or gamma (*38*), which appeared locally but failed to spread. Omicron BA.1 contains both mutations apparently driven by immune escape, K417N/T and E484A, with others, Q493R and N501Y, that compensate for loss of affinity due to the escape mutations. The effects of Q493R combine enhanced affinity for ACE2 with escape from neutralization by IGHV1-58 Abs such as G32A4. Whether the RBD mutations in Omicron BA.1 also contribute to other phenotypic changes in that variant, including tissue tropism and generally milder disease course (*39*), is not yet clear. If so, they would represent a different optimum in the evolution of the virus, potentially of advantage both to virus and to host.

The structures, binding, and neutralization data presented here show that just two or three mutations at key positions in the SARS-CoV-2 spike protein can strongly diminish the capacity of RBD-binding Abs from an unbiased panel to neutralize variants such as beta, gamma, and Omicron BA.1. Nonetheless, the example of C98C7 shows that some public-class, RBD-2 binding antibodies can retain neutralizing activity for those variants, and the data in Fig. 2 show that several more of the RBD-2 directed mAbs in the panel retained detectable binding. The mutations in the Omicron BA.1 variant that have restored ACE2 affinity loss from K417N/T and E484K/A have apparently allowed it to supplant the previously prevalent delta variant and to cause breakthrough infections in SARS-CoV-2 immune experienced individuals. In such cases, re-activation of B cell memory, for which relatively weak residual affinity appears to be sufficient, could then in principle update the repertoire by affinity maturation against the mutated antigen, as suggested by recently published studies (*40*, *41*).

Localized point mutations, which are the sources of immune escape in the beta, gamma, and Omicron BA.1 variants of SARS-CoV-2, as illustrated by the structures described here, are also responsible for immune escape of drifted influenza hemagglutin variants (*42*). In the sequence of events that has characterized successive variation of influenza-virus subtypes over the course of the past century (*43*–*45*), recalled memory, rather than activation of naive, mature B cells, has been the primary component of the response to antigenically drifted strains ((*46*–*48*). Although both memory and naive cells are present in secondary germinal centers (*49*), the re-activated and affinity-matured memory cells appear to dominate the output. Whether, upon recall, somatic hypermutation will be able to adapt antibodies in widely prevalent classes to an emerging variant or to expand and affinity mature those exemplified by the non-public class-antibodies in our panel may determine how frequently one will need to update spike-based vaccines.

## MATERIALS AND METHODS

### Study design

The aim of this study was to perform an un-biased analysis of the early memory B cell repertoire specific for SARS-CoV-2 delta and Omicron BA.1 variants. We tested 73 previously charted mAbs from COVID-19 convalescents (*2*) and determined their binding and neutralizing potency against SARS-CoV-2 variants of concern. We further characterized some of the broadly neutralizing mAbs from three key epitopic regions using cryo EM.

### Summary of Real-world mRNA vaccine effectiveness

Data for Fig. 2E came from the following references. *Clinical trial* of mRNA vaccine, with efficacy of 94-95% in preventing infection after two doses (*50*, *51*), confirmed in real-world settings (*52*–*54*). Real-world effectiveness against the *alpha variant* shown to be similar to clinical trials (*13*, *54*, *55*). Direct comparison of BNT162b2 mRNA vaccine efficacy against *alpha and beta variant infection* in Qatar during a time of approximately 50% prevalence of each (*55*) showed 89.5% and 75% efficacy against infection by alpha and beta, respectively, and over 90% for each for protection from severe outcomes (*55*). A similar comparison in the UK of the protective efficacy of two doses of the BNT162b2 mRNA vaccine for infection by *alpha and delta variants* were similar (93.7 and 88%, for alpha and delta, respectively) (*13*, *14*). Data for the Omicron BA.1 variant indicate 70% protection from severe disease after two doses of the BNT162b2 mRNA vaccine (*14*). Real-world studies allowing direct comparison of mRNA vaccine efficacy for the *gamma variant* with direct reference to another variant of concern are not yet available.

### Protein expression and purification

The expression plasmid for SARS-CoV-2 spike hexapro and RBD were gifts from Jason McLellan (*56*) and Aaron Schmidt (*57*), respectively. RBD and spike hexapro were expressed in Expi293F cells (ThermoFisher, Cat. A14527) by transfecting cells at a density of 3.0×10^6^ cells/mL with 1 μg of DNA complexed with 3 μg PEI per mL of culture. After 24 hours, glucose was added to 3 g/L and valproic acid was added to a final concentration of 3.5 mM. The conditioned medium was harvested 6 days post transfection by pelleting the cells and passing the clarified sample over Talon resin (Takara). Eluted protein was concentrated and applied to a S200 size exclusion chromatography column. RBD eluting at about 16 mL or spike eluting at about 9 mL was concentrated and used without further purification. Spike hexapro was treated with 3C protease and separated from protease and any fragments on Talon resin. Antigen binding fragments (Fab) were obtained as described (*2*).

### Cell surface binding assays

Assays for antibody binding to spike variants were described previously (*2*). Briefly, HEK 293T cells were co-transfected with plasmids encoding SARS-CoV-2 Spike (HDM-SARS2-spike-D614G-Δ21, Addgene, Cat. 158762; or HDM-SARS2-Delta variant spike-Δ21 or HDM-SARS2-Omicron BA.1 variant spike-Δ21) and GFP (pmaxGFP, Lonza) using Lipofectamine 3000 (ThermoFisher, Cat. L3000015). Delta variant spike modifications are T19R, G142D,156-157 deletion, R158G, L452R, T478K, D614G, P681R, D950N. Omicron BA.1 variant spike modifications are A67V, 69-70 deletion, T95I, G142D, 143-145 deletion, 211 deletion, L212I, 214 insertion EPE, G339D, S371L, S373P, S375F, K417N, N440K, G446S, S477N, T478K, E484A, Q493R, G496S, Q498R, N501Y, Y505H, T547K, D614G, H655Y, N679K, P681H, N764K, D796Y, N856K, Q954H, N969K, L981F. At 24 hours post-transfection, supernatant was replaced with fresh culture medium. At 48 hours, cells were detached with PBS supplemented with 2 mM EDTA. Cells were stained with 10 μg/ml of each monoclonal antibody on ice for 1 hour, then washed with FACS buffer (PBS with 2% FBS) twice. Goat anti-human IgG (Alexa Fluro 647, ThermoFisher, Cat. A21445) was the secondary antibody for detection by flow cytometry (BD Canto II). DAPi was used to distinguish dead and live cells. Spike+ cells were gated on DAPi^-^GFP^+^. Data were analyzed by FlowJo 10.7.1. The relative binding intensities of the tested mAbs for each spike variant were calculated as follows: [log10(mAb MFI) - log10(background MFI)]/ [log10(CR3022 MFI) - Log10(background MFI)] where mAb indicates the individual mAbs tested from our panel, background indicates signal from a PBS control, and CR3022 is the signal from the CR3022 mAb, which is broadly reactive to all SARS-CoV-2 variants, as a control for expression. All mAbs binding to delta or Omicron BA.1 variants were repeated at least once together with D614G.

### Pseudovirus production and neutralization assay

Pseudovirus particles were prepared as described (*2*).Spike envelope plasmid (HDM-SARS2-Delta variant spike-Δ21 or HDM-SARS2-Omicron BA.1 variant spike-Δ21), package plasmid (psPAX2, Addgene, Cat. 12260), and backbone plasmid (pLenti-CMV-Puro-LUC, Addgene, Cat. 17477) were co-transfected with Lipofectamine 3000 into HEK 293T cells (ATCC, Cat. CRL-3216). Culture medium was replaced with fresh medium at 24 hours post-transfection. Supernatants were collected at 48 hours post-transfection. Cell debris was removed by centrifugation at 300 g for 10 min before aliquoting and storing at -80°C.

Neutralization assays were performed as described (*58*). The target cell line was 293FT co-expressing human ACE2 and serine protease TMPRSS2 (provided by Marc C. Johnson, University of Missouri). Cells at 1.8 × 10^4^ cell/well were seeded in 96-well plates 16 hours before infecting. The mAbs were serially diluted, mixed with pseudovirus, and incubated at 37°C for 1 hour before adding to cells, with duplicate wells for each antibody dilution. Cells infected with pseudovirus only, but no monoclonal antibody were set as 100% infection; wells that contained cells without either pseudovirus or mAb were set as 0% infection. After 48 hours incubation at 37°C and 5% CO2, cells were processed with luminescent regent (ONE-Glo^TM^, Promega, Cat. E6120) according to manufacturer’s instructions, and luminescence (RLU) measured with a microplate reader (Biotek Synergy H1). Inhibition was calculated by 100-(RLU of mAb-RLU of blank)/ (RLU of pseudovirus -RLU of blank) ×100%. Values for 50% inhibition (IC_50_) were calculated with GraphPad Prism 9. All neutralizing mAbs were repeated at least twice with duplicated wells.

Weighted neutralization in each epitopic region was calculated by adding log_10_(IC_50_) of all neutralizing mAbs in each cluster and dividing by the counts of neutralizing mAbs in the same cluster. IC_50_ values of the Abs that lost neutralizing potency (IC_50_>50 mg/ml) against variant were set at 50 mg/ml for calculation.

### Authentic virus propagation and neutralization assay

Authentic SARS-CoV-2 viruses were propagated as described (*2*). All work with infectious SARS-CoV-2 was performed under Biosafety Level-4 conditions at the National Emerging Infectious Diseases Laboratories (NEIDL). Briefly, viruses (USA-WA1/2020 or Omicron BA.1 B.1.1.529) were amplified in NR‐596 VeroE6 cells (BEI Resources) by infection at an approximate MOI of 0.001 PFU/cell in DMEM + 2% heat-inactivated-FBS (Gibco). Infected cells were observed daily for progression of cytopathic effect (CPE). Supernatant was collected and clarified by centrifugation. Stocks was adjusted to 10% final concentration prior to store at -80°C. Stocks were confirmed mycoplasma negative from DNA using the MycoSEQ detection system (ThermoFisher) and Endotoxin levels were determined <0.2 EU/mL using the Lonza QCL-1000 endpoint chromogenic LAL assay.

Viral neutralization reduction assays: An Avicel plaque reduction assay was used to quantify plaques. One day prior to the assay, 2 ml of VeroE6 cells were seeded at a density of 8.0 × 10^5^ cells per well of a 6-well plate (Falcon Polystyrene Microplates, Cat. 353934). On the day of infection, Abs were diluted at two-fold serial dilutions with Dulbecco’s Phosphate Buffered Saline (DPBS)(Gibco) and prepared in triplicate and plated in triplicate per antibody. 1000 plaque forming units/ml (PFU/ml) of SARS-CoV-2 were incubated with each dilution and DPBS control and then incubated at 37°C/5% CO_2_ for 1 hour. DPBS alone without virus was used as background control and SARS-CoV-2 incubated with DPBS was used as 100% infection control. The maintenance medium was removed prior to add 200 μL of mixture of antibody and viruses or controls to the pre-seeded Vero E6 cells and incubated for 1 hour at 37°C/5% CO_2_ with gentle rocking every 10-15 min to prevent monolayer drying. At the meantime, the overlay was prepared by mixing by inversion Avicel 591 overlay (DuPont Nutrition & Biosciences, Wilmington, DE) and 2X Modified Eagle Medium (Temin’s modification, Gibco) supplemented with 2X antibiotic‐antimycotic (Gibco), 2X GlutaMAX (Gibco) and 10% FBS in a 1:1 ratio. After 1 hour, 2 mL of overlay was added to each well and the plates was incubated for 48 hours at 37°C/5% CO_2_. Infected cells were then fixed using 10% neutral buffered formalin and then stained with 0.2% aqueous Gentian Violet (RICCA Chemicals, Arlington, TX) in 10% neutral buffered formalin for 30 min, followed by rinsing and plaque counting. The half maximal inhibitory concentrations (IC_50_) were calculated using GraphPad Prism 9. All neutralizing mAbs were tested in three separate dilutions with triplicate wells per dilution. Results are in Fig. S6.

### Biolayer interferometry (BLI)

BLI measurements were carried out with a Sartorius BLItz. RBD with a C-terminal his-tag was titrated to determine a loading concentration that avoided saturating the Ni-NTA sensor, which required loading 20 to 40 μg/mL over 60 s. We used association and dissociation steps of 90 s and a series of Fab concentrations between 16 μM to 62.5 nM, after control experiments had confirmed that loading 1000 nM Fab did not bind the sensor tip non-specifically. BLI sensorgrams were processed to remove data from the initial steps and allow the association step to begin at zero seconds. The BLI data were fit by non-linear regression using a modified “association then dissociation” equation in Graphpad Prism 6 software to allow for baseline changes by adding a constant to the dissociation phase of the equation. The “Koff” and “Kon” parameters were globally constrained. The fitted parameters *K_d_*_,_ *k_on_*, and *k_off_,* with associated error, and overall goodness of fit, are listed in Table S4.

### Cryo-EM structure determination

Grids containing SARS-CoV-2 spike hexapro in complex with Fabs were prepared as described. Briefly, spike was combined with a 3-fold molar excess of Fab to a final protein concentration of 0.7 mg/mL, applied to thick C-flat 1.2/1.3 400 mesh Cu grids, and cryo-plunged with a Gatan CP3. Grids were imaged with a Titan Krios 300 keV microscope equipped with a Gatan K3 direct electron detector by automated low-dose imaging with Serial EM (*59*). Details of data collection are in Table S2.

Software and hardware used to process micrographs were configured and maintained by SBGrid. All data processing was performed in RELION (*60*, *61*). Beam-induced motion correction of micrograph movies was performed with UCSF MotionCor2 (*62*) followed by contrast transfer function estimation with CTFFIND-4.1 (*63*), both implemented in RELION. Particles were picked from motion corrected micrographs by crYOLO using a general model (*64*). Particles were extracted with 4-fold down sampling and subjected to 2D and 3D classification in RELION. We obtained 3D classes that reached the down sampled Nyquist-limited resolution, re-extracted these subsets of particles at the original pixel size, and subjected them to 3D auto refinement in RELION. Complexes of G98C7 and G32Q4 were C3 symmetric with the three Fab-bound RBD in an “up” conformation; spike in complex with G32A4 had one RBD each in “up”, “down”, and “out” conformations and lacked overall threefold symmetry.

In all cases, the Fab was poorly resolved at high resolution in the full particle reconstructions, necessitating local refinement. C3-symmetric, full-particle stacks were symmetry expanded, and a new particle stack was extracted centered on the Fab-epitope interface and with a smaller box size. In the case of G32A4, only the Fab bound to the RBD in the down conformation was well resolved, and a particle stack was extracted centered on this Fab-epitope interface. After reconstruction of the particle stacks, 3D autorefinement was carried out, in some cases several rounds with progressively tighter masks. We then used 3D classification to remove both noisy subparticles and RBD without bound Fab. Subsequent rounds of 3D autorefinement with a tighter mask followed by sharpening with RELION yielded ~4 Å reconstructions for each RBD-Fab complex. Maps involved in key 3D processing decisions are in Fig. S2, and details of image processing are in Table S2. The angular distribution plots in Fig. S7 show sampling of most of the angular space, despite varying degrees of preferential orientation.

Models of the Fab-RBD complexes were prepared by identifying the most similar heavy and light chain in the protein data bank (PDB IDs: for C98C7, 7CH4 and 7CDI; for G32A4, 7MLZ; for G32Q4, 7BEL and 7JXE). The coordinates were docked as rigid bodies into cryo-EM densities along with an RBD fragment (6YZ5). Residues in the template structures were altered to the actual sequences in coot (*65*). Initial fitting of the densities was performed with ISOLDE, and refinement, with phenix refine (*66*, *67*). We used PISA (https://pubmed.ncbi.nlm.nih.gov/17681537/) to identify residues at the interface between ACE2 (PDB ID: 6M0J) or each of the three mAbs reported here and the SARS-CoV-2 RBD.

## Supplementary Materials

### Other Supplementary Material for this manuscript includes the following:

MDAR Reproducibility Checklist
