# Supplementary material for: Antibodies induced by ancestral SARS-CoV-2 strain that cross-neutralize variants from Alpha to Omicron BA.1
Source: Sci Immunol. 2022 May 10:eabo3425. doi: 10.1126/sciimmunol.abo3425 (PMC9097876; doi:10.1126/sciimmunol.abo3425)
Supplement: Supplementary file 1 — Figs. S1 to S7 Tables S1 to S4 [file sciimmunol.abo3425_sm.pdf]

Science Immunology
AAAS

Supplementary Materials for

Antibodies induced by ancestral SARS-CoV-2 strain that cross-neutralize variants from Alpha to Omicron BA.1

Ian W. Windsor *et al.*

Corresponding authors: Duane R. Wesemann, dwesemann@bwh.harvard.edu;
Stephen C. Harrison, harrison@crystal.harvard.edu

DOI: 10.1126/sciimmunol.abo3425

The PDF file includes:

Figs. S1 to S7
Tables S1 to S4

Other Supplementary Material for this manuscript includes the following:

MDAR Reproducibility Checklist
Table S5

Antibodies induced by ancestral SARS-CoV-2 strain that cross-neutralize variants from Alpha to Omicron BA.1

Ian W. Windsor, Pei Tong, Olivia Lavidor, Ali Sanjari Moghaddam, Lindsay G.A. McKay, Avneesh Gautam, Yuezhou Chen, Elizabeth A. MacDonald, Duck Kyun Yoo, Anthony Griffths, Duane R. Wesemann, Stephen C. Harrison

Content	Page
Table of Contents	S1
Figure S1. Representative histograms of mAb binding to cell-surface spike	S2
Figure S2. Cryo-EM image processing and 3D reconstruction	S3
Figure S3. Map resolution and map-model Fourier shell correlation plots	S4
Figure S4. RBD recognition by broadly neutralizing SARS-CoV-2 Abs	S5
Figure S5. BLI sensorgrams and non-linear regression analysis summarized in Table S4	S6
Figure S6. Neutralization of authentic SARS-CoV-2 viruses by mAbs characterized in this paper	S7
Figure S7. Angular distribution plots for cryo-EM reconstructions	S8-9
Table S1. Neutralization of SARS-CoV-2 variant pseudotypes	S10
Table S2. Cryo-EM data and final model statistics	S11
Table S3. Ig gene segments for RBD-2 mAbs in the panel in Figure 2	S12
Table S4. Association and dissociation rate constants and equilibrium dissociation constants for Fab binding with RBD of SARS-CoV-2 variants of concern	S13
Table S5. Raw data for Figure 2 and Figure S5	

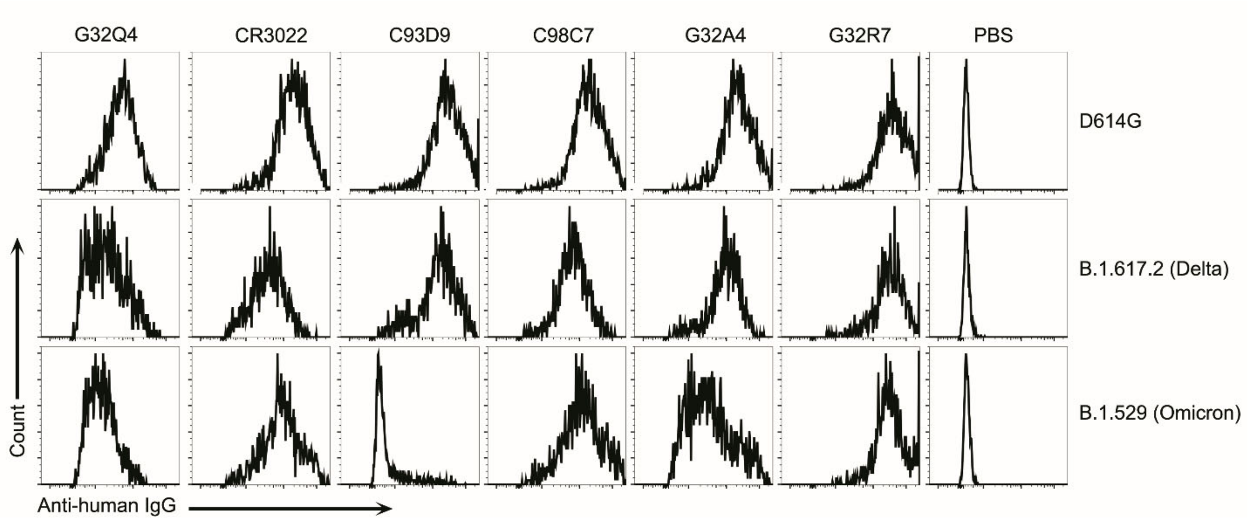

Figure S1. Representative histograms of mAb binding to cell-surface spike. Each row of the plot is the indicated spike expressed on the HEK 293T cell surface. Each column is the indicated monoclonal antibody or PBS control.

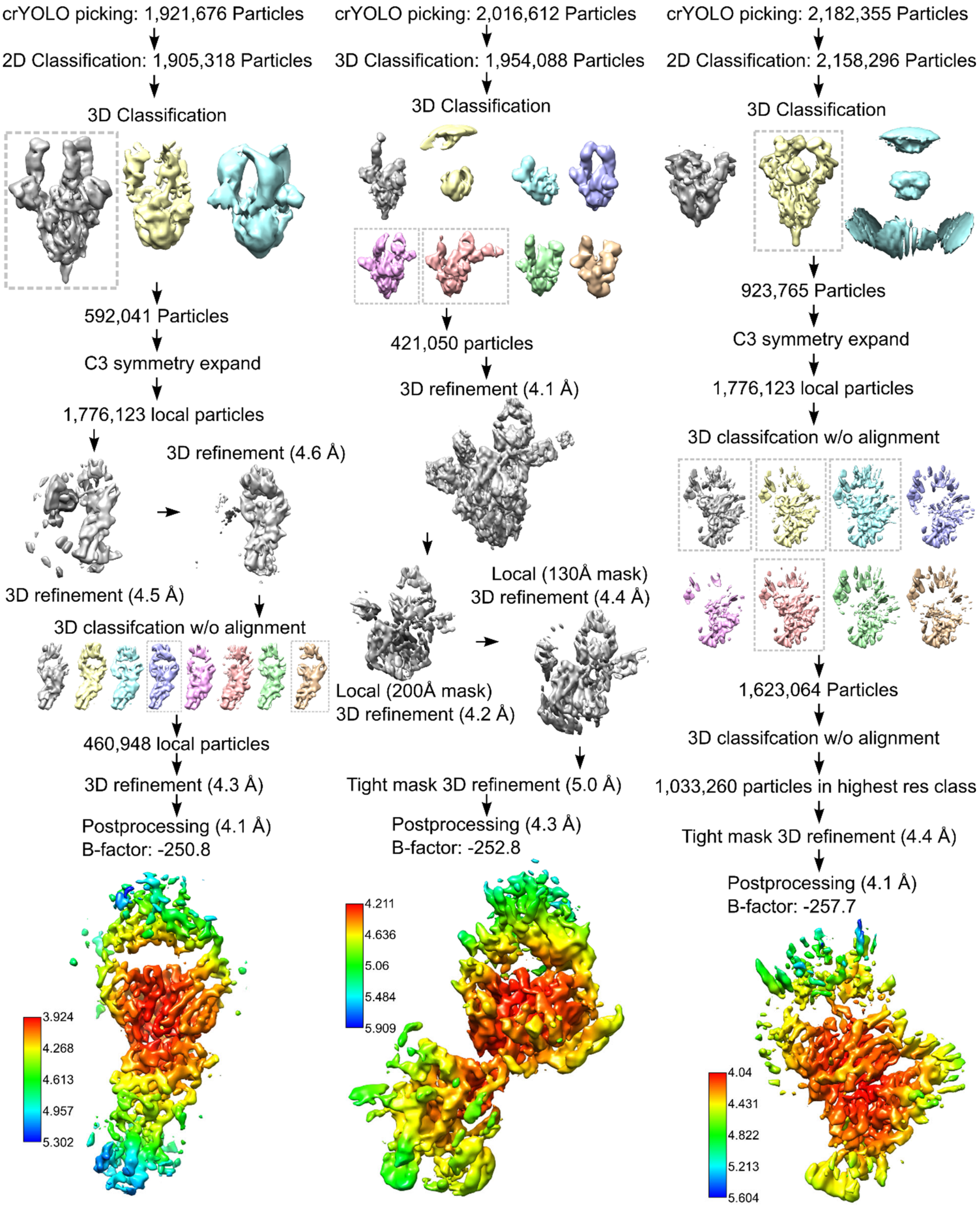

Figure S2. Cryo-EM image processing and 3D reconstruction. Processing schemes for local reconstructions of C98C7 (left), G32A4 (middle), and G32Q4 (right) Fabs in complex with the SARS-CoV-2 RBD. Final reconstructions that are deposited in the EMDB are shown at the bottom and colored according to local resolution.

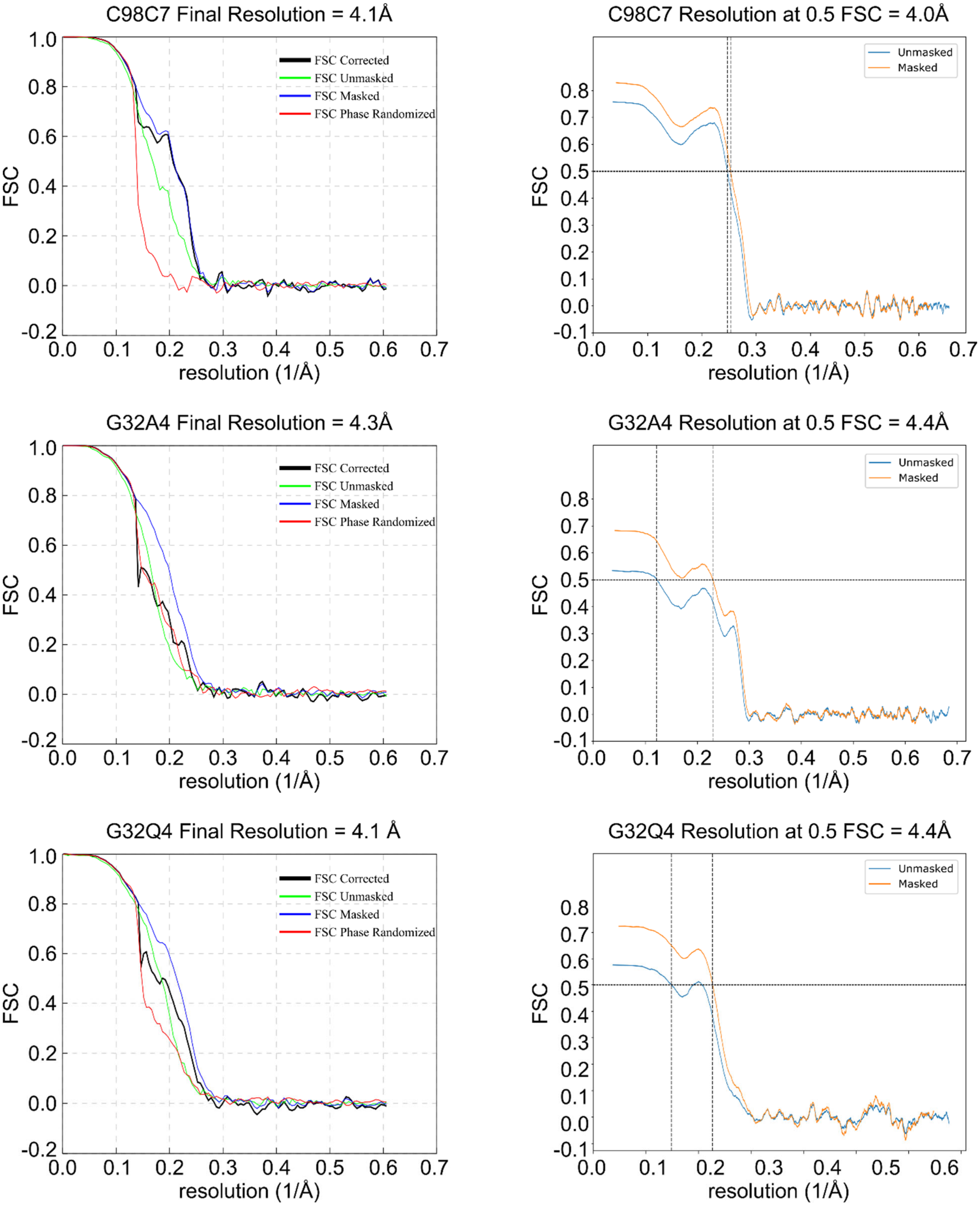

Figure S3. Map resolution and map-model Fourier shell correlation plots. Left. FSC plots generated by the RELION 3D autorefine protocol. Right. Map-model FSC plots generated by phenix.refine.

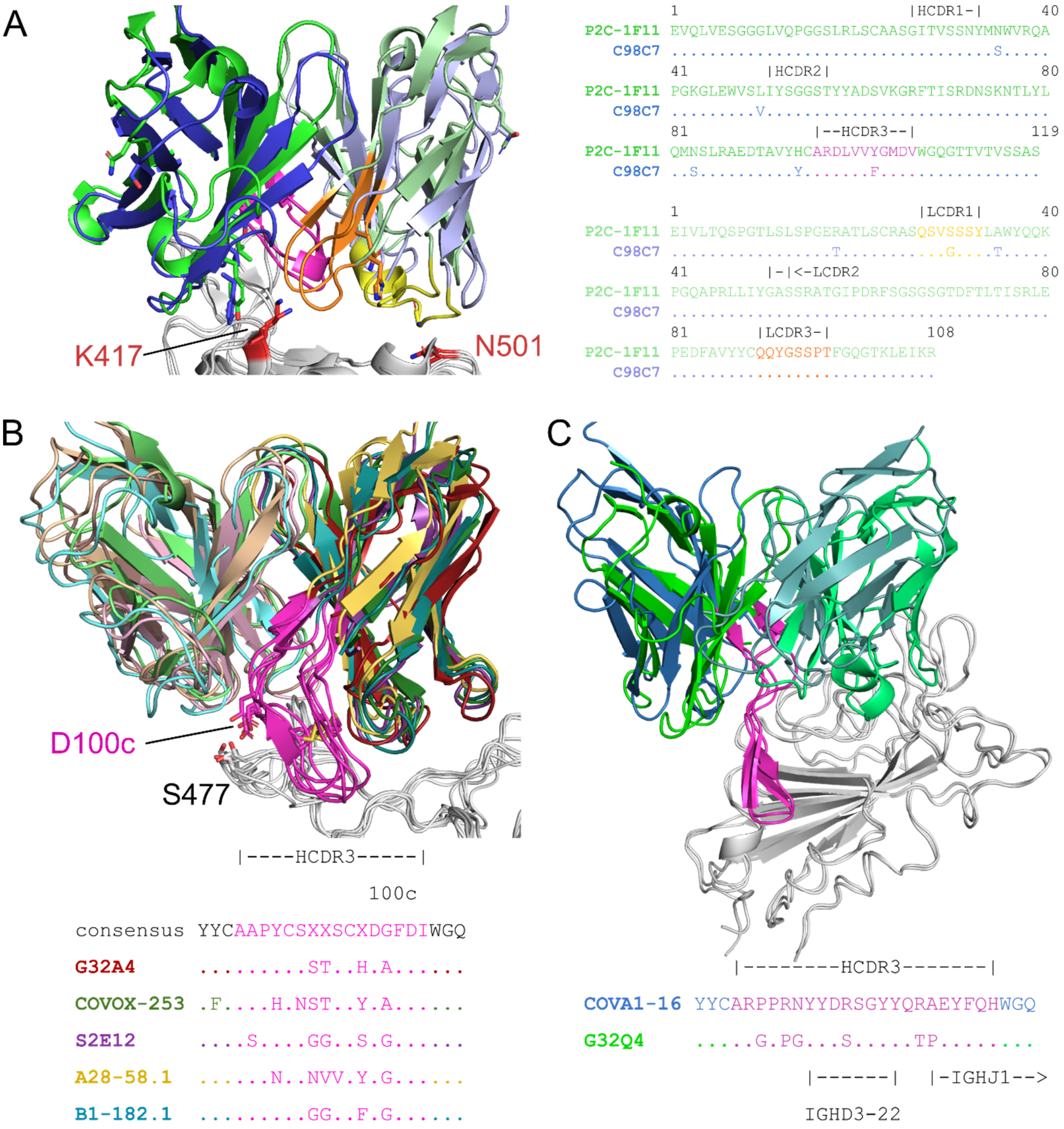

Figure S4. RBD recognition by broadly neutralizing SARS-CoV-2 Abs. Complexes of RBD and related antibodies are aligned on the RBD for: (A) C98C7 and P2C-1F11 (PDB ID: 7CDI), (B) G32A4 and VH1-58 mAbs (PDB IDS: 7BEN, 7R6X, 7LRS, and 7MLZ), and (C) G32Q4 and COVA1-16 (PDB ID: 7JMW). Key conserved elements are colored as shown in the sequence alignments.

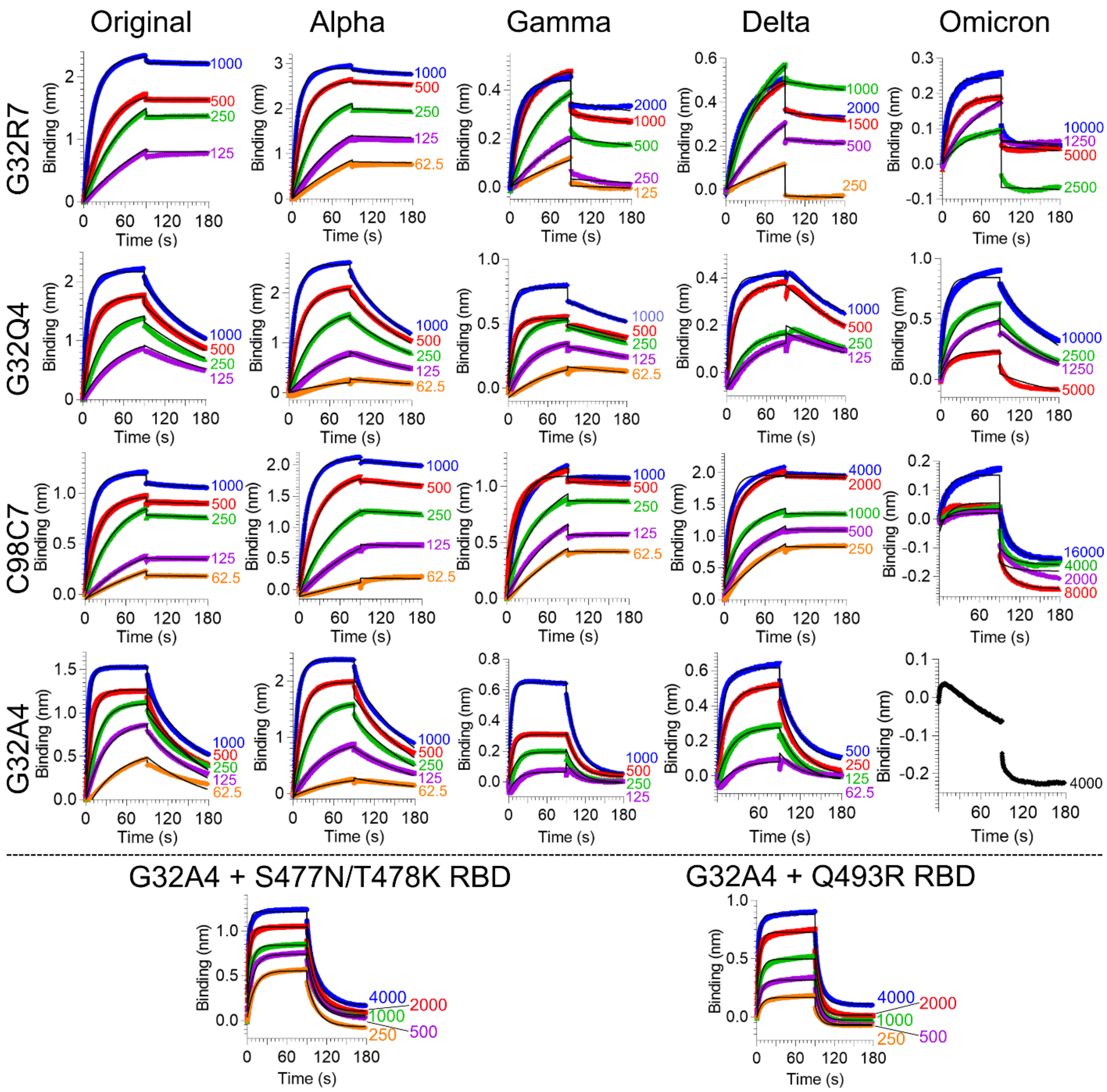

Figure S5. BLI sensorgrams and non-linear regression analysis summarized in Table S4. Raw data were fit to obtain values of k_{on}, k_{off}, and K_d. These values, with standard error and goodness of fit, are reported in Table S4.

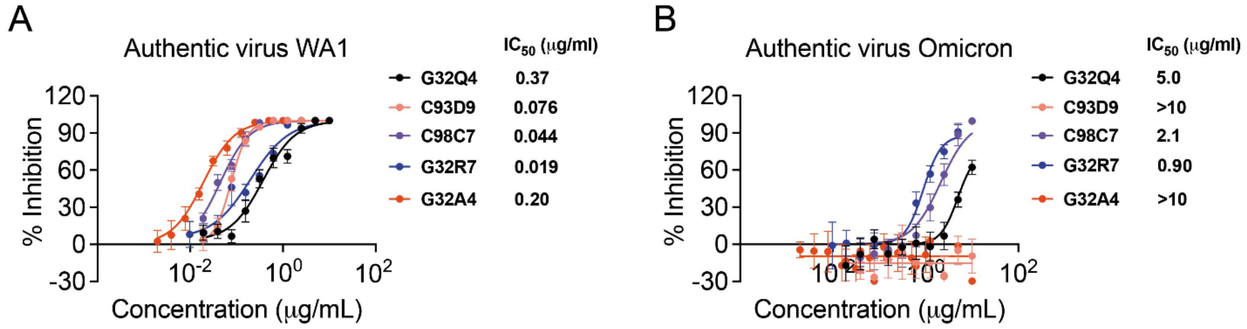

Figure S6. Neutralization of authentic SARS-CoV-2 viruses by mAbs characterized in this paper. (A) Authentic WA1 virus neutralization profiles for 5 antibodies. (B) Authentic Omicron variant neutralization profiles for 5 antibodies.

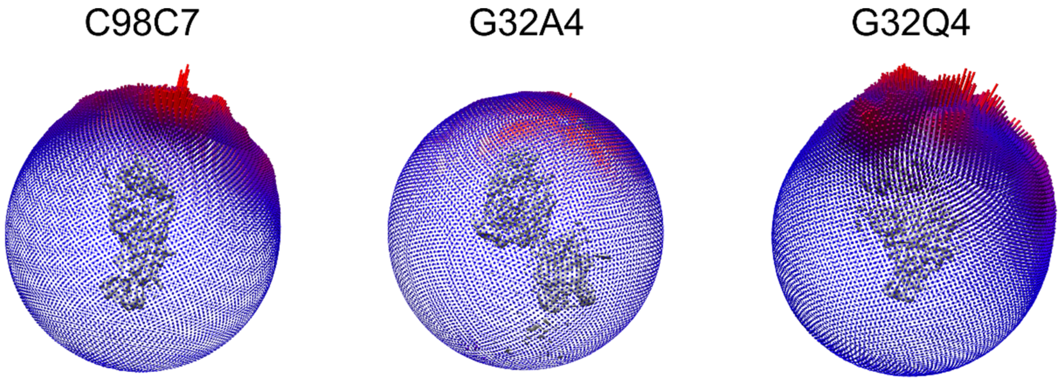

Figure S7. Angular distribution plots for cryo-EM reconstructions. Plots were generated in Chimera using the output files from Relion 3D autorefinement. RBD-Fab complexes are orientated like the maps depicted at bottom of Figure S1.

Table S1. Neutralization of SARS-CoV-2 variant pseudotypes.

Clone ID	IC50 (μg/ml) Delta	IC80 (μg/ml) Delta	IC50 (μg/ml) Omicron	IC80 (μg/ml) Omicron
C89A10	10.200	22.820	>50	>50
C63C7	14.460	>50	>50	>50
C63D3	38.430	>50	>50	>50
G32Q4	0.316	1.181	6.666	11.650
C30C2	>50	>50	>50	>50
C93D9	0.045	0.128	>50	>50
C98C7	0.023	0.070	1.067	3.772
G32A4	0.003	0.008	>50	>50
G32B6	0.012	0.036	>50	>50
C12A2	0.017	0.053	>50	>50
G32A8	0.070	0.294	>50	>50
G32C4	5.235	24.050	>50	>50
C15D10	28.610	>50	>50	>50
C18C11	>50	>50	>50	>50
C15D4	>50	>50	>50	>50
C102A4	>50	>50	>50	>50
C53F11	>50	>50	31.510	>50
C53F9	>50	>50	>50	>50
C53E1	10.030	36.140	6.406	15.030
C53F10	>50	>50	>50	>50
C41P1	16.440	31.180	>50	>50
C12E7	10.260	13.030	~ 14.54	17.200
G32Q3	>50	>50	>50	>50
C12D5	>50	>50	>50	>50
G32B1	8.766	18.750	14.640	39.920
C63C8	4.156	9.418	>50	>50
C18C10	>50	>50	>50	>50
C12C10	>50	>50	>50	>50
C93D1	>50	>50	>50	>50
C12B10	>50	>50	>50	>50
C12E9	>50	>50	>50	>50
C12C6	>50	>50	>50	>50
C29C2	>50	>50	>50	>50
C12D1	1.186	2.965	>50	>50
G32R7	1.851	7.898	0.162	0.753
C163E5	>50	>50	>50	>50
G32Q2	>50	>50	>50	>50
C12D6	>50	>50	>50	>50
C83A7	>50	>50	>50	>50
C53E4	>50	>50	>50	>50
C53E6	>50	>50	>50	>50

C83B6	>50	>50	>50	>50
C41N3	>50	>50	>50	>50
C93D6	>50	>50	>50	>50
C12C11	>50	>50	>50	>50
C12C9	>50	>50	>50	>50
C41C3	>50	>50	>50	>50
C83A8	>50	>50	>50	>50
G32A12	>50	>50	>50	>50
C12D8	>50	>50	>50	>50
C83B5	>50	>50	>50	>50
C12A8	>50	>50	>50	>50
C63D4	>50	>50	>50	>50
C18D5	>50	>50	>50	>50
G32Q1	>50	>50	>50	>50
C7A9	>50	>50	>50	>50
C15C3	>50	>50	>50	>50
C7A4	>50	>50	>50	>50
C882C12	>50	>50	>50	>50
C30A11	>50	>50	>50	>50
C83B8	>50	>50	>50	>50
C83A10	>50	>50	>50	>50
C102A1	>50	>50	>50	>50
C163D1	>50	>50	>50	>50
C12B2	>50	>50	>50	>50
C107A2	>50	>50	>50	>50
G32R10	>50	>50	>50	>50
C41N6	>50	>50	>50	>50
C30A5	>50	>50	>50	>50
C163E6	>50	>50	>50	>50
C163E11	>50	>50	>50	>50
C107A1	>50	>50	>50	>50
C12B3	>50	>50	>50	>50

Table S2. Cryo-EM data and final model statistics

Complex	C98C7-RBD	G32A4-RBD	G32Q4-RBD
PDB	7swo	7swn	7swp
EMDB	25477	25479	25478
Microscope	Titan Krios	Titan Krios	Titan Krios
Voltage(kV)	300	300	300
Detector	Gatan K3	Gatan K3	Gatan K3
Magnification (nominal)	105,000	105,000	105,000
Energy filter slit width	20 eV	20 eV	20 eV
Calibrated pixel size (Å/pix)	0.825	0.825	0.825
Exposure rate (e- /pix/sec)	20.144	20.323	20.404
Frames per exposure	50	50	50
Total electron exposure ($e^-/Å^2$)	52.2	52.7	52.9
Exposure per frame ($e^-/Å^2$)	1.044	1.053	1.058
Defocus range (µm)	-0.6, -1.8	-0.6, -1.8	-0.6, -1.8
Automation software	Serial EM	Serial EM	Serial EM
# of Micrographs used	13233	15693	15246
Particles extracted	1921676	2016612	2182355
Total # of refined particles	460948	421050	1033260
Symmetry imposed	C1	C1	C1
Estimated accuracy of translations/rotations	1.17/2.89	1.35/2.93	1.25/3.03
Map sharpening B-factor	-250.8	-252.8	-252.8
Unmasked Resolution at 0.5/0.143 FSC (Å)	5.9/4.3	6.1/4.8	4.7/4.0
Masked resolution at 0.5/0.143 FSC (Å)	4.8/4.1	6.5/4.3	5.2/4.1
Model refinement and validation			
Amino acids	627	422	436
RMSD bond lengths (Å)	0.007	0.008	0.008
Angles (°)	1.18	1.28	0.916
Mean B-factors	79.6	90.1	166
Ramachandran Favored	90.0	78.1	87.9
Allowed	10.0	21.9	12.1
Outliers	0	0	0
Rotamer Outliers	0.19	0	0
Clash score	25.6	40.5	39.5
C-beta outliers	0	0	0
CaBLAM outliers	4.7	8.1	3.5
CC (mask)	0.76	0.64	0.72
MolProbity score	2.45	2.85	2.68

Table S3. Ig gene segments for RBD-2 mAbs in the panel in Figure 2.

mAb	VH	DH	JH	VL	JL
C30C2	IGHV3-66*01	N/A	IGHJ3*02	IGKV1-5*03	IGKJ2*01
C93D9	IGHV3-53*01	IGHD2-15*01	IGHJ4*02	IGKV1-5*03	IGKJ2*01
C98C7	IGHV3-66*01	IGHD3-22*01	IGHJ6*02	IGKV3-20*01	IGKJ1*01
G32A4	IGHV1-58*01	IGHD2-2*01	IGHJ3*02	IGKV3-20*01	IGKJ1*01
G32B6	IGHV1-2*02	IGHD3-9*01	IGHJ4*02	IGLV2-8*01	IGLJ3*02
C12A2	IGHV1-2*02	IGHD3-10*01	IGHJ6-03	IGLV2-14*01	IGLJ2*01
G32A8	IGHV1-2*02	IGHD3-16*01	IGHJ4*02	IGLV2-8*01	IGLJ3*02
G32C4	IGHV5-51*01	IGHD5-18*01	IGHJ4*02	IGLV2-14*01	IGLJ1*01
C15C10	IGHV6-1*01	IGHD6-6*01	IGHJ6*02	IGLV2-14*01	IGLJ1*01
C18C11	IGHV3-30-3*01	IGHD3-10*01	IGHJ4*02	IGKV1-39*01	IGKJ4*01
C15D4	IGHV4-34*01	IGHD2-21*01	IGHJ6-03	IGKV1-39*01	IGKJ5*01

Table S4. Association and dissociation rate constants and equilibrium dissociation constants for Fab binding with RBD of SARS-CoV-2 variants of concern.

RBD	Parameter	G32R7	C98C7	G32A4	G32Q4
Original	K_d (nM)	[a] <0.1	2.8	53	57
	k_{on} ($M^{-1}s^{-1}$)	6.5×10^5	9.8×10^5	1.9×10^6	1.2×10^6
	k_{off} (s^{-1})	$< 10^{-5}$	2.8×10^{-4}	1.0×10^{-2}	6.6×10^{-3}
	R^2	0.9996	0.9997	0.9936	0.9976
Alpha	K_d (nM)	2.8	5.5	60	75
	k_{on} ($M^{-1}s^{-1}$)	1.1×10^6	7.5×10^5	1.4×10^6	9.3×10^5
	k_{off} (s^{-1})	3.0×10^{-4}	4.1×10^{-4}	8.2×10^{-4}	7.0×10^{-3}
	R^2	0.9994	0.9994	0.9979	0.9991
Gamma	K_d (nM)	[a] <0.1	1.1±0.4	360	13
	k_{on} ($M^{-1}s^{-1}$)	4.6×10^6	7.0×10^5	1.5×10^6	2.0×10^6
	k_{off} (s^{-1})	$< 10^{-5}$	$(7.9 \pm 2.9) \times 10^{-5}$	5.5×10^{-4}	2.5×10^{-3}
	R^2	0.9963	0.9924	0.9980	0.9979
Delta	K_d (nM)	50	3.5 ± 0.9	290	53
	k_{on} ($M^{-1}s^{-1}$)	1.6×10^5	2.8×10^5	1.2×10^6	1.2×10^6
	k_{off} (s^{-1})	7.9×10^{-4}	$(9.9 \pm 2.6) \times 10^{-4}$	3.4×10^{-2}	6.2×10^{-3}
	R^2	0.9996	0.9974	0.9928	0.9915
Omicron	K_d (nM)	68	22,000	N.A.	1800
	k_{on} ($M^{-1}s^{-1}$)	1.0×10^5	2.4×10^3		7.9×10^3
	k_{off} (s^{-1})	7.1×10^{-4}	5.5×10^{-2}		1.4×10^{-2}
	R^2	0.9982	0.9934		0.9984
S477N/T478K	K_d (nM)	N.D.	N.D.	790	N.D.
	k_{on} ($M^{-1}s^{-1}$)			8.2×10^5	
	k_{off} (s^{-1})			6.4×10^{-2}	
	R^2			0.9973	
Q493R	K_d (nM)	N.D.	N.D.	3,700	N.D.
	k_{on} ($M^{-1}s^{-1}$)			3.5×10^5	
	k_{off} (s^{-1})			1.3×10^{-1}	
	R^2			0.9978	

Standard errors > 10% of the fitted parameter are explicitly reported. [a]K_d and k_{off} values poorly determined due to very slow dissociation. These values reported as thresholds in the main text. N.A., not applicable, binding too weak to measure. N.D., not determined.
